# Evaluating the Knowledge, Attitudes and Practices of Medical Laboratory Professionals Towards Implementing Enterprise Risk Management in Harare, Zimbabwe: A Cross‐Sectional Study

**DOI:** 10.1002/hsr2.70931

**Published:** 2025-06-16

**Authors:** Donald Vhanda, Judy Mwenje, Reynold Vhanda, Kudzai Chinowaita, Itai James Blessing Chitungo, Tafadzwa Dzinamarira

**Affiliations:** ^1^ Chemical Pathology Unit, Department of Laboratory Diagnostic and Investigative Sciences Faculty of Medicine and Health Sciences University of Zimbabwe Harare Zimbabwe; ^2^ Graduate Business School Bindura University of Science Education Zimbabwe; ^3^ Department of Marketing National University of Science and Technology Zimbabwe; ^4^ Department of Actuarial Sciences National University of Science and Technology Zimbabwe; ^5^ ICAP at Columbia University Harare Zimbabwe

**Keywords:** attitude, enterprise risk management, knowledge, medical laboratory, practice

## Abstract

**Background and Aims:**

Medical laboratories play a crucial role in healthcare, with the majority of medical judgements based on clinical laboratory testing outcomes. Therefore, laboratories must provide accurate, timely, reliable, and interpretable diagnostic results that inform patient care. However, laboratory operations are characteristically vulnerable to complex and interdependent risks, including errors in testing, biosafety and biosecurity breaches, equipment malfunctions, regulatory and compliance issues, and financial concerns. Enterprise Risk Management (ERM) is a holistic approach that identifies, assesses, mitigates these risks and identifies opportunities to ensure patient safety, laboratory quality, and organisational sustainability. This study evaluated the knowledge, attitudes, and practices (KAP) of the medical laboratory professionals towards the implementation ERM in Harare, Zimbabwe.

**Methods:**

The study design was cross‐sectional, involving 41 questionnaires and interviews which were self‐administered to participants in the medical laboratories in Harare, Zimbabwe. The questionnaires assessed the knowledge, attitude and practice of ERM implementation by interrogating known ERM elements. Statistical Package for Social Sciences (SPSS) version 22 was used for data analysis.

**Results:**

ERM culture and ERM best practices were strongly correlated (0.735). Crobach's alpha coefficients of 0.89 and 0.92 were obtained showing internal consistency. Six out of nine ERM hard aspects were missing at the time of assessment. The mean score for the soft aspects of ERM was 3.3 and the risk culture rating was 2.81 out of a scale of 5.

**Conclusion:**

The risk culture did not support ERM implementation. The knowledge, attitude and practices show risk immaturity. ERM knowledge gaps exist among medical laboratory professionals. The study highlights the need for targeted training and awareness programs to enhance ERM knowledge and attitude among laboratory professionals.

## Summary

- **What this study adds**
  ∘ The study demonstrated the significance of assessing the KAP of medical laboratory professionals towards implementing ERM.
  ∘ The findings highlight the need for targeted interventions to enhance ERM awareness, understanding, and adoption.
  ∘ ERM knowledge gaps exist among medical laboratory professionals.

**Abbreviations**
ERM: Enterprise Risk Management
ISO: International Organisation for Standardisation
RM: Risk Management
SHEQ: Safety Health Environment and Quality

## 1 Introduction

Medical laboratories play a crucial role in healthcare, with over 70% of medical decisions based on laboratory testing outcomes [1, 2, 3]. Therefore, medical laboratories need to provide accurate, timely, reliable, and interpretable diagnostic results that inform patient care. However, laboratory operations are characteristically vulnerable to complex and interdependent risks including errors in testing, biosafety and biosecurity breaches, equipment malfunctions, regulatory and compliance issues, and financial concerns [4, 5, 6]. Enterprise Risk Management (ERM) is a holistic approach that identifies, assesses, and mitigates these risks and identifies opportunities to ensure patient safety, laboratory quality, and organisational sustainability [7, 8, 9]. ERM is a systematic approach that aligns processes, people, technology, knowledge and strategy with the purpose of evaluating, and managing the risks and opportunities faced by an organisation or enterprise in an integrated and coordinated manner [5, 7].

Risk management (RM) is a critical component of quality assurance in clinical laboratory systems and ISO15189:2022 standard, section 8.5 [3, 10]. Implementing RM in an enterprise‐wide approach makes it cheaper, more comprehensive, inclined towards seizing opportunities, and more strategic for business entities as opposed to the traditional or ‘silo’ approaches [7, 9, 11]. This involves anticipating what could go wrong, assessing the frequency and severity of occurrences, and coming up with mitigatory measures to lessen the impact of risks to tolerable levels [2].

Despite its demonstrated importance in various sectors over the years, and reference in the ISO 15189:2022 and ISO 31000 standard, ERM implementation in medical laboratories remains limited [3, 12, 13, 14]. Some of the reasons for limited implementation include lack of resources, lack of adequate training, lack of structures that support ERM implementations, and the complexity of laboratory operations [3, 9, 15]. Medical laboratory professionals’ KAP toward ERM are key factors influencing its adoption and implementation.

This study aims to evaluate the KAP of medical laboratory professionals towards ERM in Harare, Zimbabwe. Understanding the KAP levels informs the policymakers in making targeted interventions, planning for quality improvement initiatives, and training to close the identified gaps and enhance patient outcomes.

## 2 Methods

### 2.1 Study Design, Population, and Setting

A cross‐sectional survey design was used to assess the KAP of medical laboratory professionals towards implementing ERM. The study targeted professionals working in public and private medical laboratories in Harare, Zimbabwe. The population included laboratory managers, senior laboratory scientists, laboratory technicians, quality assurance officers, laboratory nurses, and laboratory hands. Forty one self‐administered questionnaires and interviews were distributed and conducted in May 2018 using a convenience sampling approach to target laboratories that were either accredited or working to attain ISO 15189 accreditation since these were the ones most probable to be implementing ERM according to Beasley and Branson (2005) who argue that “large organisations are more likely to report complete ERM processes” [16]. Therefore, these were used as case studies upon which the implementation of ERM KAPs were studied. The exclusion criteria also included employees with less than 1 year of experience, students and interns, and non‐laboratory professionals.

### 2.2 Data Collection

A self‐administered questionnaire and interview guide were used to gather data. The questionnaire consisted of sections asking for demographic information (age, sex, working experience, and education), questions asking about the knowledge, attitude, and practices in a yes/no format, and on a 1–5 Likert scale. Respondents were given questionnaires to answer on their own; those who wanted to seek further clarity on any section of the questionnaire or study were given time to ask as needed. This was done to generate as representative information as possible. Questionnaires were self‐administered because we used a purposive stratified sampling approach, so as to target the key informants, to enable the signing of consent forms, and also to conduct the interviews at the end. This was done to cost‐effectively utilise resources and time. The questionnaire was developed based on available literature and extracted from best practices from the ISO 31000 standard [12, 17]. The researchers drafted, discussed and agreed on the questions in the questionnaire and interview guide. The questions were ranked using a Likert scale of 1–5, some were semi‐structured to allow respondents to detail out their responses. The interview guide was used to probe the participants further on selected questions. Data was collected and cleaned to ensure completeness and consistency.

### 2.3 ERM Elements

The core elements of an effective ERM framework, as defined by the Committee of Sponsoring Organisations of the Treadway Commission (COSO), include objective setting and strategy, communication, performance, governance and culture and, information, and reporting structures [17]. Twenty elements that define these core aspects were drafted in the questionnaire (Appendix 1) to assess and determine the ERM practice and culture within the organisations. The ERM elements can further be divided into the ‘hard’ and ‘soft’ elements. The “hard” aspects focus on quantifiable, structured risks and processes, while “soft” aspects encompass cultural, behavioural, and communication elements that influence risk management effectiveness [3, 17]. The hard aspects of risk interrogated for in this study as guided by the ISO 15189 and ISO 31000 standard best practices included checking for the RM policy, RM common language document, RM committee, central risk function, RM training and retraining, risk registers, reporting structure, risk appetite and the change management policy. The soft aspects asked in the questionnaire (see Appendix 1) examined the organisational ERM culture, behaviour, and the RM communications aspects.

### 2.4 Scoring Criteria

For the hard elements, a yes or no responses were used to indicate the presence or absence of a structural or process element respectively. For the ERM elements whose responses were ranked on a Likert scale of 1–5, the mean scoring previously used before was applied to interpret the practices in the medical laboratories as shown below [3].

Mean scores (M) ranging from 1.0 ≤ M < 1.8: Very poor practice

Mean scores ranging from 1.8 ≤ M < 2.6: Poor practice

Mean scores ranging from 2.6 ≤ M ≤ 3.4: Neutral

Mean scores ranging from 3.4 < M ≤ 4.2: Good practice

Mean scores ranging from 4.2 < M ≤ 5.0: Very good practice [3].

### 2.5 Validity and Reliability

For validity of the data collection tools, an expert review and also pilot testing (to 10 people) was done before the questionnaires were administered to the actual study participants. The other reason for piloting was to check if the questionnaires were easy to understand and also to estimate the time that was required to go through the questionnaire. The Cronbach's alpha coefficient was used to measure the internal consistency and reliability of scales in the questionnaire responses. Table 1 below summarises the coefficients for ERM practices and culture.

**Table 1:** Reliability statistics.

	Cronbach's coefficient (alpha)	No. of items
ERM best practices	0.923	21
Risk culture	0.887	20

*Note:* Summary of the Crobach's alpha of the study.

### 2.6 Data Analysis

Data was checked for accuracy and completeness. Analysis and data presentation were done using the Statistical Package for Social Sciences (SPSS) version 22 (SPSS Inc., Chicago, IL, USA). Frequency distributions and percentages were used to analyse demographic data. Mean scores were used for knowledge, attitude, and practice scores. A bivariate correlation coefficient was also generated to assess the link between ERM culture and ERM practices.

### 2.7 Ethical Approvals and Consent to Participate

The Bindura University of Science Education Ethics and Bio‐Safety Research Committee gave ethical approval for this study. Participating organisations authorised their staff members to participate in the study. Informed consent was sought from the prospective participants before enrolment. Participants were free to withdraw at any stage of the study. To maintain patient confidentiality in the study, all identifying information was anonymised and replaced with unique codes, and access to the data was restricted to authorized personnel only.

## 3 Results

A total of 41 questionnaires were returned and 41 interviews were conducted with the study participants. Table 2 below summarises the study participants' characteristics.

**Table 2:** Participants’ demographic characteristics.

Characteristic	Number (*n*)	Proportion (%)
Total	*n* = 41	100%
Sex: Male	25	61.6
Age (years)		
20–30	19	46.3
> 30–40	20	48.8
> 40	2	4.9
**Work categories**		
Laboratory management	9	22
Laboratory scientists	13	31.7
Laboratory technicians	7	17.1
Laboratory nurses	6	14.6
Laboratory hands	6	14.6
**Work experience (years)**		
< 5	21	51.2
5–10	19	46.3
> 10 < 15	1	2.5

The respondents' work experience was crucial for identifying experienced individuals with proper understanding of the laboratory systems. Most of the respondents (n = 21; 51.2%) were in the less than 5 years category. The medical laboratories in Harare were dominated by the 30–40 years age group.

### 3.1 ERM Best Practice

The findings were summarised into hard aspects (Table 3) and soft aspects (Table 4) of ERM as shown below. Presence or absence of the hard aspects of ERM was assessed by a yes or no responses respectively in Table 3.

**Table 3:** Frequency of responses on the hard aspects of ERM best practice.

ERM best practice components	(Yes) %	(No) %
1. Documented RM policy	80.5	19.5
2. Risk common language document	43.9	56.1
3. RM committee	46.3	53.7
4. Central risk function well‐qualified in RM	41.5	58.5
5. RM training in the previous 1 year	48.8	51.2
6. Risk registers	70.7	29.3
7. Formal reporting of risks to superior	78.0	22.0
8. Risk appetite	39.0	61.0
9. Change management policy	43.9	56.1
**Overall score**	**54.7**	**45.3**

*Note:* Summary of questionnaire responses to the ERM best practice hard aspects.

**Table 4:** Mean scores on the soft aspects of ERM best practice responses.

ERM best practice components	Mean
1. Policy that supports the goals and objectives of RM	3.65
2. RM policy understanding	3.68
3. RM policy clarity	3.36
4. Risk ownership assignment at all levels	3.15
5. Use of indicators and risk dashboards	3.38
6. Continuous risk identification	3.59
7. Risk impact analysis	3.54
8. Regular communication of risk status	3.12
9. Regular update of the RM log	3.10
10. Identification and communication of risks	3.46
11. Conduct of risk status meetings	2.24
12. Management full consideration risk in determining the best course of action	3.34
**Overall score**	**3.30**

*Note:* (The following scales are used to assess the respondents' views of the ERM best practice infrastructure.

Mean scores ranging from 1.0 ≤ M < 1.8: Very poor practice

Mean scores ranging from 1.8 ≤ M < 2.6: poor practice

Mean scores ranging from 2.6 ≤ M ≤ 3.4: Neutral

Mean scores ranging from 3.4 < M ≤ 4.2: Good practice

Mean scores ranging from 4.2 < M ≤ 5.0: Very good practice) [3].

Only 3 out of the 9 hard aspects were in place across the sampled laboratories. The 3 aspects that were generally present were RM policy, risk registers and the provision of reporting structures to the superiors. The questionnaire and interview revealed that the organisations use different policies such as the quality manual, safety manual, Safety Health Environment and Quality (SHEQ) manual. The researchers discovered that those who claimed to have a RM policy were actually referring to the safety and quality manuals.

The ERM practice soft aspects mean scores are shown in Table 4 below.

From Table 4, only 5 out of the possible 12 ERM soft aspects that were investigated showed good practice (mean scores above of 3.4 and above). The overall mean score of 3.3 falls in the neutral category indicating that the practice in the medical laboratory sector falls way below the desired grade, though it is an indication that some RM practices are going on. That coupled with a large absence of ERM hard aspects point to risk immaturity.

**Figure 1:**
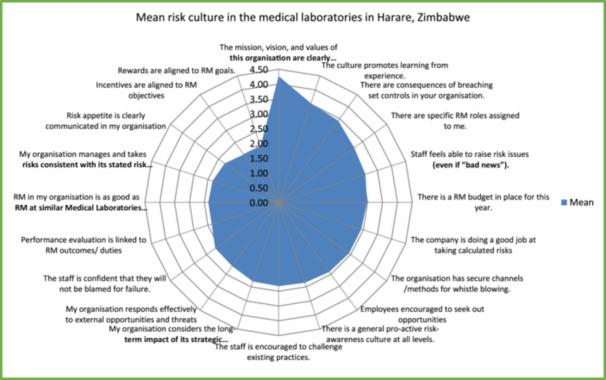
The radar scale showing the mean risk culture among medical laboratories in Harare *‐ (The following scale were used to evaluate ERM culture in the medical laboratories. Mean scores ranging from 1.0 ≤ M < 1.8: Very poor practice, Mean scores ranging from 1.8 ≤ M < 2.6: poor practice, Mean scores ranging from 2.6 ≤ M ≤ 3.4: Neutral, Mean scores ranging from 3.4 < M ≤ 4.2: Good practice, Mean scores ranging from 4.2 < M ≤ 5.0: Very good practice) [3].

The questionnaires were also used to assess the organisations' ERM culture using Likert scales of 1**–**5 and the mean scores of the 20 ERM culture elements are shown in Figure 1 below.

The radar scale shows poor mean risk culture in the medical laboratories in Harare, as indicated by a rating of 2.81 on a scale of 1–5 for all the elements.

ERM culture and ERM best practices were strongly correlated with a coefficient of 0.735.

## 4 Discussion

In this study respondents were asked questions to evaluate their KAP towards ERM in Harare, the capital city of Zimbabwe. There was a strong positive correlation between ERM culture and ERM best practices (0.735). The overall mean score for the ERM soft aspects was 3.3 and the overall risk culture rating was 2.81. The findings provide valuable insights into the state of ERM awareness and adoption among the laboratory professionals.

### 4.1 Risk Management Policy

The bigger fraction of study participants 33 (80.5%) had RM policies. This finding suggests that the industry was doing some form of RM, but on further interogations through interviews, it was discovered that the policies were integrated into quality assurance. This is in line with authors who stated that RM is not new to clinical laboratories, but only formal RM is new [2, 4]. Respondents were asked to produce the RM policy during the interviews and majority produced the quality policy manual. That mix‐up was a critical pointer to the need for staff training in the sector [9].

### 4.2 Risk Common Language

A bigger portion 23 (56%) of respondents did not have the risk common language documents in place. Where there is good RM practice, people speak the same RM language. The lack of a risk common language document was also found to be an obstacle in ERM implementation by Renault (2016) [18]. The absence of risk departments, committees, and designated RM personnel in the majority of organisations under study was the main reason why most of the ERM hard aspects were missing as shown in Table 3. On analysing responses from the same organisation it showed that personnel at different levels gave conflicting responses on the same questions, indicating information asymmetry. At one organisation the laboratory practitioner said *“the issues that you are asking for are handled by the quality assurance office, if you want accurate responses you are better off taking your questionnaires to that department.”*

### 4.3 Risk Management Training

Most respondents, 21 (51.2%), had not received RM training in the preceding year. From the interviews, some people had not received any form of RM training ever since they joined their organisations. One laboratory nursing staff member at one private institution asked, *“where do we find such trainings on risk management, is it from workshops, short courses or diplomas? I have never been exposed to such trainings. Maybe if you talk to our management they will plan something for us*.” Only the quality assurance trainings were popular among the respondents due to resource limitations, understandable for organisations working towards accreditation. However, respondents concurred in interviews that the risk awareness trainings were critical in improving RM in organisations. Most organisation had the risk function bunched into quality assurance, with only one organisation reporting having a risk manager and a risk officer. Rostami et al (2015) and Beasley (2022) highlighted the need to overcome the lack of training barrier to implement ERM well [9, 19]. Reza et al (2016) reported that education and training for RM are critical success factors [20].

### 4.4 Risk Management Expertise

From the study, only one organisation had a standalone RM department. The rest incorporated RM duties into the SHEQ departments. This concurs with findings from Yaraghi et al (2011) who stated that organisational structure can be a challenge in ERM practice [21]. In a study from the United Kingdom, a lack of experienced personnel was reported as a challenge to RM implementation in small and medium enterprises [19]. Lack of expertise was also noted in a study by Vhanda et al, (2024) [3].

### 4.5 Risk Ownership

The survey results show a low average score of 3.15 for RM ownership across all levels and types of risks. This could stem from the absence of clear policies that define how to manage risks in a coordinated manner. Most of the respondents were unaware of their own risk responsibilities. During the interviews, many questions were directed to the quality assurance department, indicating that quality assurance units were perceived as the main risk owners for the respective organisations. A laboratory hand supervisor at a government laboratory indicated that *“we don't own any risks in this department, we report everything to the SHEQ department and they are the ones responsible.”* Assigning RM roles to individuals is essential in ensuring accountability of the process. Previous studies by Rostami et al (2015) and Mustapha et al (2015) have identified the lack of ownership as a major challenge in RM [19, 22].

### 4.6 Communication

Effective ERM implementation requires good communication. The interviews revealed that only two organisations had a simple and transparent way of sharing risk information, even when it was negative*. “You cannot take bad news to the boss unless if you want to fire yourself from this organisation. In our reports we have to carefully craft what he likes; otherwise you will be embarrassed in front of subordinates,”* remarked one laboratory manager from a private laboratory. That was also exhibited by a low mean score of 3.1. In most institutions, risks were not reported freely; and with victimisation tendencies in some cases, against the person who would have raised the issue. Delayed feedback from reports was also a common phenomenon in some institutions. *“We always report these issues in meetings, but no one comes back with feedback”*, complained a laboratory practitioner at one laboratory. Mustapha et al. (2015) also emphasized the importance of communication in ERM implementation, where employees should make the developments part of their daily activity [22]. ISO 31000 defines communication as ongoing and iterative processes, top‐down and up, that an organisation does to provide, share, or obtain information and to engage in dialogue regarding ERM [12, 17].

### 4.7 Risk Appetite and Tolerances

Risk appetite is defined as the extent of risk an organisation is willing to accept in pursuit of value [3]. Many organisations did not communicate their risk appetite clearly, and the average score of 2.24. This could be due to the absence of proper documents that defined their risk appetites. About 60% of the respondents showed that they did not have such documents. Also, the institutions did not take risks consistently with the specified risk appetite as shown by a low average score of 2.34, meaning that laboratories did not adequately assess the potential concerns of the risks they faced, exposing their organisations to potential business operations disruption. These findings were also supported by the absence of RM policies in most laboratories.

### 4.8 ERM Soft Aspects

Seven pillars out of twelve were below the expected ranges in the sector showing a deficiency of ERM soft aspects. The study compared the RM practices in the medical laboratory sector in Zimbabwe with the ISO 31000 guidelines and found that the ERM implementation in the industry was immature [11, 17]. This finding was consistent with a survey by several authors who reported that ERM was underdeveloped and immature in various organisations [7, 16]. If ERM is immature it will not provide strategic value for the enterprise.

### 4.9 ERM Culture in the Medical Laboratories In Harare

The correlation between ERM implementation and risk culture was found to be strong as exhibited by 73.5%. The result shows that institutions with strong risk cultures tend to implement efficient RM systems that follow international best practices. This result agrees with the views of several authors who argue that a robust risk culture is a crucial factor for effective ERM implementation [9, 15, 19, 23]. The mean culture rating of 2.81 indicates poor culture in the industry. It is critical for the institution to have a risk culture that supports the implementation of ERM. The ERM implementation program depends on the organisational culture, which should be integrated into the corporate strategy and then reflected in the daily operations of the organisation. The implementation of ERM is not only influenced by the quality of RM policies or resources, but also by their suitability for the purpose. Hence, the correct fit of organisational culture is essential for achieving ERM best practices.

This exploratory study unearthed some key insights into the ERM practices in medical laboratories in Harare. The Cronbach coefficient alphas of 0.923 and 0.887 showed that results can be generalised to the whole country since the samples were drawn from public and private organisations. These can guide the regulatory authorities and policymakers to come up with targeted ERM interventions to improve patient outcomes and compliance in medical laboratories.

## 5 Limitations

The self‐reported data by the study participants may introduce some bias to the KAP findings. Convenience sampling approaches may limit generalisability. A small sample size of the study in one geographical town of Zimbabwe may also introduce some selection bias. The other limitation of this study is that it did not assess the risk of failure of the medical devices used in the laboratories.

## 6 Recommendations

The authors recommend further studies with a bigger sample size that is more representative of laboratories in the country. The interviews may also be done by trained interviewers and not by the researcher to exclude researcher bias in the study. With more resources, it would be better to include a bigger and more representative sample of laboratories in the country. Further research can be done to explore the laboratory specific ERM KAPs and the risks of failure of medical devices used in the clinical laboratory settings.

## 7 Conclusions

The ERM culture did not support proper ERM implementation. Medical laboratories in Harare, Zimbabwe showed risk immaturity, because there are gaps between practice and the expected international best practices in ERM implementation. The study highlights the strong need for enhanced ERM awareness, trainings and adoption among laboratory professionals in Harare, Zimbabwe. Addressing the knowledge and structural gaps, cultivating the right culture could improve the ERM implementation, contributing to safer and more effective medical laboratory services. There is need for further targeted and more comprehensive studies to understand the impact of ERM in the medical laboratories.

## Author Contributions

**Donald Vhanda:** conceptualization, investigation, visualization, validation, methodology, formal analysis, resources, data curation, writing – original draft, writing – review and editing, project administration. **Judy Mwenje:** methodology, supervision, writing – review and editing, conceptualization. **Reynold Vhanda:** investigation, software, formal analysis, data curation, writing – review and editing. **Kudzai Chinowaita:** methodology, writing – review and editing, formal analysis, software, data curation, conceptualization. **Itai James Blessing Chitungo:** writing – original draft, writing – review and editing, methodology, validation. **Tafadzwa Dzinamarira:** writing – original draft, writing – review and editing, visualization, validation.

## Conflicts of Interest

The authors declare no conflicts of interest.

## Transparency Statement

The lead author Donald Vhanda affirms that this manuscript is an honest, accurate, and transparent account of the study being reported; that no important aspects of the study have been omitted; and that any discrepancies from the study as planned (and, if relevant, registered) have been explained.

## Supporting information

Studies RNYN.

## Data Availability

The datasets used and/or analysed during the current study are available from the corresponding author on reasonable request.

## References

[1] K. A. Sikaris , “Enhancing the Clinical Value of Medical Laboratory Testing,” Clinical Biochemist Reviews 38, no. 3 (November 2017): 107–114. 29332975 PMC5759162

[2] D. R. Eliza and D. Minodora , “Risk Management in Clinical Laboratory: From Theory to Practice,” Acta Medica Marisiensis 61, no. 4 (December 2015): 372–377.

[3] D. Vhanda , K. Chinowaita , F. Chinowaita , et al., “Enterprise Risk Management Implementation Challenges in Medical Laboratories in Harare, Zimbabwe,” Health Science Reports 7, no. 9 (2024): e70088. 39319251 10.1002/hsr2.70088 PMC11420288

[4] S. W. Njoroge and J. H. Nichols , “Risk Management in the Clinical Laboratory,” Annals of Laboratory Medicine 34, no. 4 (July 2014): 274–278. 24982831 10.3343/alm.2014.34.4.274 PMC4071183

[5] N. Saleh , O. Gamal , M. A. A. Eldosoky , and A. R. Shaaban , “An Integrative Approach to Medical Laboratory Equipment Risk Management,” Scientific Reports 14, no. 1 (February 2024): 4045. 38374369 10.1038/s41598-024-54334-z PMC10876531

[6] A. M. Osman , W. I. Al‐Atabany , N. S. Saleh , and A. M. El‐Deib, Decision Support System for Medical Equipment Failure Analysis. In: 2018 9th Cairo International Biomedical Engineering Conference (CIBEC). 2018. 94–7.

[7] I. J. Dabari and S. Z. Saidin , “A Theoretical Framework on the Level of Risk Management Implementation in the Nigerian Banking Sector: The Moderating Effect of Top Management Support,” Procedia ‐ Social and Behavioral Sciences 164 (2014): 627–634.

[8] A. P. Liebenberg and R. E. Hoyt , “The Determinants of Enterprise Risk Management: Evidence From the Appointment of Chief Risk Officers,” Risk Management and Insurance Review 6, no. 1 (2003): 37–52.

[9] M. S. Beasley and B. C. Branson , An Overview of Enterprise Risk Management Practices (Poole College of Management, 2022), 13th ed., 3–5.

[10] R. T. Jansen , D. Kenny , V. Blaton , et al., “Usefulness of EC4 Essential Criteria for Quality Systems of Medical Laboratories as Guideline to the ISO 15189 and ISO 17025 Documents,” Clinical Chemistry and Laboratory Medicine 38, no. 10 (June 2005): 1057–1064. 10.1515/CCLM.2000.158 11140624

[11] M. Ciorciari and P. Blattner , “Enterprise Risk Management Maturity‐Level Assessment Tool,” Society of Actuaries 41 (2008): 1–25.

[12] O. Guzel and E. I. Guner , “Iso 15189 Accreditation: Requirements for Quality and Competence of Medical Laboratories, Experience of a Laboratory I,” Clinical Biochemistry 42, no. 4 (March 2009): 274–278. 19863920 10.1016/j.clinbiochem.2008.09.011

[13] H. Elamir , “Enterprise Risk Management and Bow Ties: Going Beyond Patient Safety,” Business Process Management Journal 26 (August 2019): 770–785.

[14] N. Saleh and A. Abo Agyla , “An Integrated Assessment System for the Accreditation of Medical Laboratories,” Biomedical Engineering/Biomedizinische Technik 66, no. 1 (February 2021): 107–114. 10.1515/bmt-2019-0133 32598292

[15] C. Kanhai and L. Ganesh , “Factors Influencing The Adoption Of Enterprise Risk Management (ERM) Practices by Banks in Zimbabwe,” International Journal of Business and Commerce 3, no. 6 (2014): 01–17.

[16] M. S. Beasley , R. Clune , and D. R. Hermanson , “Enterprise Risk Management: An Empirical Analysis of Factors Associated With the Extent of Implementation,” Journal of Accounting and Public Policy 24, no. 6 (November 2005): 521–531.

[17] D. Gjerdrum and M. Peter , “The New International Standard on the Practice of Risk Management–A Comparison of ISO 31000: 2009 and the COSO ERM Framework,” Risk Management 31, no. 21 (2011): 8–12.

[18] B. Y. Renault , J. N. Agumba , and O. A. Balogun , “Drivers for and Obstacles to Enterprise Risk Management in Construction Firms: A Literature Review,” Procedia Engineering 164 (2016): 402–408.

[19] A. Rostami , J. Sommerville , I. L. Wong , and C. Lee , “Risk Management Implementation in Small and Medium Enterprises in the UK Construction Industry,” Engineering, Construction and Architectural Management 22, no. 1 (2015): 91–107.

[20] M. R. Hosseini , N. Chileshe , J. Jepson , and M. Arashpour , “Critical Success Factors for Implementing Risk Management Systems in Developing Countries,” Construction Economics and Building 16, no. 1 (2016): 18–32.

[21] N. Yaraghi and R. G. Langhe , “Critical Success Factors for Risk Management Systems,” Journal of Risk Research 14, no. 5 (2011): 551–581.

[22] M. Mustapha and A. Adnan , “A Case Study of Enterprise Risk Management Implementation in Malaysian Construction Companies,” International Journal of Economics and Financial Issues 5, no. 1 (2015): 70–76.

[23] J. R. S. Fraser and B. J. Simkins , “The Challenges of and Solutions for Implementing Enterprise Risk Management,” Business Horizons 59, no. 6 (2016): 689–698.

