# Supplementary material for: Evaluating the Knowledge, Attitudes and Practices of Medical Laboratory Professionals Towards Implementing Enterprise Risk Management in Harare, Zimbabwe: A Cross‐Sectional Study
Source: Health Sci Rep. 2025 Jun 16;8(6):e70931. doi: 10.1002/hsr2.70931 (PMC12168490; doi:10.1002/hsr2.70931)
Supplement: Supplementary file 1 — Studies RNYN. [file HSR2-8-e70931-s001.docx]

**Appendix 1: Questionnaire**

**INSTRUCTIONS:**

1. Please answer all questions fully and honestly.
2. Where boxes are provided indicate your answer by ticking the appropriate box.
3. Do not write your name or identity on the questionnaire.

**SECTION 1: PERSONAL DETAILS**

1. Gender?

| Male |  | Female |  |
| --- | --- | --- | --- |

1. Age group?

| 20 - 30 years |  | 31-39 years |  | 40 years and above |  |
| --- | --- | --- | --- | --- | --- |

1. How long have you been employed at your organisation?

| 1–5 years |  | 6–10 years |  | 11– 15 years |  | 16– 20 years |  | 20 years and above |  |
| --- | --- | --- | --- | --- | --- | --- | --- | --- | --- |

**Section 2: ERM Best Practices**

Please tick the appropriate response in the boxes provided on the extent to which your organisation practises the following?

| **Question** | | | | | Yes | | No | |  |
| --- | --- | --- | --- | --- | --- | --- | --- | --- | --- |
| 1. Does your organisation have a documented risk management policy? | | | | |  | |  | |  |
| 1. Is there a risk common language document | | | | |  | |  | |  |
| 1. Is there a risk management committee | | | | |  | |  | |  |
| 1. Is the central risk function well qualified in RM? | | | | |  | |  | |  |
| 1. Did you receive any RM training since 2017? | | | | |  | |  | |  |
| 1. Are risk registers used at your organisation? | | | | |  | |  | |  |
| 1. Do you formally report any risks to your superior? | | | | |  | |  | |  |
| 1. Do you have documented limits that restrict the level of risk that you can take (Risk appetite) | | | | |  | |  | |  |
| 1. Is there a documented Change Management Policy? | | | | |  | |  | |  |
|  | Statement | SD | D | N | | A | | SA | |
|  | The policy support the goals and objectives of risk management |  |  |  | |  | |  | |
|  | I understand risk management policy |  |  |  | |  | |  | |
|  | The risk management policy is well expressed and explained |  |  |  | |  | |  | |
|  | Risk ownership assigned at all levels for all risks |  |  |  | |  | |  | |
|  | There is use of monitoring methodologies such as key risk indicators and risk dashboards |  |  |  | |  | |  | |
|  | Risks are continuously identified at my organisation |  |  |  | |  | |  | |
|  | The potential impacts of the identified risks areanalysed |  |  |  | |  | |  | |
|  | The risk status and changes in the level or overall organisation’s risks are communicated regularly at my organisation |  |  |  | |  | |  | |
|  | Are employees and stakeholders educated on RM and encouraged to actively identify communicate risks |  |  |  | |  | |  | |
|  | The risk management log updated on regular basis at my organisation |  |  |  | |  | |  | |
|  | Management fully consider risk in determining the best course of action |  |  |  | |  | |  | |
|  | Monthly risk audits are carried out in my department |  |  |  | |  | |  | |

**SECTION 3-RM Implementation challenges**

Do you think your institution is likely to face these challenges in implementing RM Framework?

Please tick the appropriate response in the boxes provided.

**Key: Strongly Disagree (SD); Disagree (D); Neutral (N); Agree (A); Strongly Agree (SA);**

| **QUESTION** | RATING | | | | |
| --- | --- | --- | --- | --- | --- |
|  | SD | D | N | A | SA |
| 1. Increased workload |  |  |  |  |  |
| 1. Lack of financial resources to implement the program |  |  |  |  |  |
| 1. Lack of RM expertise within the organisation to implement the program. |  |  |  |  |  |
| 1. Lack of support from senior management. |  |  |  |  |  |
| 1. people is an area posing big challenge |  |  |  |  |  |
| 1. Failure by leaders to spearhead the project |  |  |  |  |  |
| 1. timeliness of information is a problem, |  |  |  |  |  |
| 1. lack of information needed, |  |  |  |  |  |
| 1. over-regulation in organization hinder RM implementation, |  |  |  |  |  |
| 1. strong competition from other type of management techniques to be implemented, |  |  |  |  |  |
| 1. wide discrepancy between expectation and practices in RM implementation, |  |  |  |  |  |
| 1. inadequate technology support (i.e. installation of information technology system for risk identification and assessment), |  |  |  |  |  |
| 1. organization structure deters RM implementation, |  |  |  |  |  |
| 1. Insufficient necessary level of investment for ERM implementation |  |  |  |  |  |

**Section 4: Risk Culture**

Please tick the appropriate response in the boxes provided

| Statement | SD | D | N | A | SA |
| --- | --- | --- | --- | --- | --- |
| 1. There is a general pro-active risk-awareness culture at all levels. |  |  |  |  |  |
| 1. The staff is encouraged to challenge existing practices. |  |  |  |  |  |
| 1. Staff feels able to raise risk issues (even if “bad news”). |  |  |  |  |  |
| 1. Employees encouraged to seek out opportunities |  |  |  |  |  |
| 1. The staff is confident that they will not be blamed for failure. |  |  |  |  |  |
| 1. The culture promotes learning from experience. |  |  |  |  |  |
| 1. Rewards are aligned to RM goals. |  |  |  |  |  |
| 1. Risk appetite is clearly communicated in my organisation |  |  |  |  |  |
| 1. My organisation responds effectively to external opportunities and threats |  |  |  |  |  |
| 1. My organisation manages and takes risks consistent with its stated risk appetite |  |  |  |  |  |
| 1. My organisation considers the long-term impact of its strategic decisions on its risk appetite |  |  |  |  |  |
| 1. The mission, vision, and values of this organisation are clearly communicated |  |  |  |  |  |
| 1. The company is doing a good job at taking calculated risks |  |  |  |  |  |
| 1. RM in my organisation is as good as RM at similar Medical Laboratories in the country |  |  |  |  |  |
| 1. Incentives are aligned to RM objectives |  |  |  |  |  |
| 1. There are consequences of breaching set controls in your organisation. |  |  |  |  |  |
| 1. Performance evaluation is linked to RM outcomes/ duties |  |  |  |  |  |
| 1. The organisation has secure channels /methods for whistleblowing. |  |  |  |  |  |
| 1. There are specific RM roles assigned to me. |  |  |  |  |  |
| 1. There is a RM budget in place for this year. |  |  |  |  |  |

**Open ended questions**

1. What do you see as your organisation’s 3 greatest strengths in risk

management?..........................................................................................................................................................................................................................................................................................................................................................................................................................................

2. What do you see as your organisation’s 3 greatest weaknesses in risk management?............................................................................................................................................................................................................................................................................................................................................................................................................................................

3. What do you believe are 3 ways of improving the current risk management practices in your organisation?.................................................................................................................................................................................................................................................................................................................................................................................................................................................................

**INTERVIEW GUIDE**

1. What are the risks to which the organisation is exposed?
2. How would you comment on the effectiveness of the risk governance structures in place?
3. What would you want changed to improve the culture of the organisation towards RM?
4. How do you distinguish between risks and opportunities in conducting your daily tasks?
5. How do you describe the commitment and the demonstration of the organisation to uphold good RM practice?
6. How easy is it for you to communicate any risk issues in the organisation? Explain?
7. Are there any incentives for individuals who manage their risks well? If yes, what are these?
8. Are there references materials in place that makes the entire organisation have a uniform understanding of RM in the organisation?
